# Statistical evaluation of the effectiveness of dual amplitude-gated stereotactic body radiotherapy using fiducial markers and lung volume

**DOI:** 10.1016/j.phro.2022.10.001

**Published:** 2022-10-06

**Authors:** Yoshinori Tanabe, Hidekazu Tanaka

**Affiliations:** a Department of Radiological Technology, Graduate School of Health Sciences, Okayama University, 5-1 Shikata-cho, 2-chome, Kita-ku, Okayama-shi, 700-8558, Japan; b Department of Radiation Oncology, Yamaguchi University Graduate School of Medicine, 1-1-1 Minamikogushi, Ube, Yamaguchi 755-8505, Japan

**Keywords:** SBRT, Stereotactic body radiotherapy, 4DCT, Four-dimensional computed tomography, AP, Anterior–posterior, LR, Left–right, SI, Superior–inferior, CGC, Center of gravity coordinates, Fiducial marker, Respiratory gating method, Stereotactic body radiotherapy, Tumor tracking, Lung cancer, 4DCT

## Abstract

**Background and purpose:**

The low tracking accuracy of lung stereotactic body radiotherapy (SBRT) risks reduced treatment efficacy. We used four-dimensional computed tomography (4DCT) images to determine the correlation between changes in fiducial marker positions and lung volume for lung tumors, and we evaluated the effectiveness of the combined use of these images in lung SBRT.

**Materials and methods:**

Data of 30 patients who underwent fiducial marker placement were retrospectively analyzed. We calculated the motion amplitudes of the center of gravity coordinates of the lung tumor and fiducial markers in each phase and the ipsilateral, contralateral, and bilateral lung volumes using 4DCT. Moreover, we calculated the cross-correlation coefficient between the fiducial marker position and the lung volume changes waveform for the motion amplitude waveform of the lung tumor over three gating windows (all phases, ≤2 mm^3^, and ≤3 mm^3^).

**Results:**

Compared with the lung volume, approximately 30 % of the fiducial markers demonstrated a low correlation with the lung tumor. In the ≤2 mm^3^ and ≤3 mm^3^ gating windows, the cross-correlation coefficients between the lung tumor and the optimal marker (r > 0.9: 83 % and 86 %) were significantly different for all fiducial markers (r > 0.9: 39 %, 53 %) and the ipsilateral (r > 0.9: 35 % and 40 %), contralateral (r > 0.9: 44 % and 41 %), and bilateral (r > 0.9: 39 % and 45 %) lung volumes.

**Conclusions:**

Some of the fiducial markers showed a low correlation with the lung tumor. This study indicated that the combined use of lung volume monitoring can improve tracking accuracy.

## Highlights

• Approximately 30% of the fiducial markers demonstrated a low correlation on comparing lung volumes.
• Monitoring of lung volume can achieve stable tracking of lung tumors.
• Dual monitoring by employing the marker and lung volume may possibly avoid the deterioration of monitoring accuracy.

## 1 Introduction

Various methods are available for motion management in lung stereotactic body radiotherapy (SBRT) to achieve safe and accurate treatment [1], [2]. A real-time tumor-tracking system for SBRT has been developed using the multileaf collimator tumor-tracking technique, and this system involves magnetic resonance imaging and a gating system that uses internal fiducial markers and spirometer-monitored techniques [2], [3]. Notably, this real-time tumor-tracking system for SBRT enables the tracking of the tumor and respiratory movements [3], [4].

The real-time tumor-tracking system with fiducial markers and fluoroscopic imaging can also be used to accurately treat lung tumors by monitoring fiducial markers within several millimeters of the gating window [5]. However, the monitoring accuracy of this system may be reduced because of dropouts and potential migration of fiducial markers during treatment [6], [7].

The respiratory movement of lung tumors varies with location (i.e., upper and middle lung, lower lung lobe, diaphragm, and near the heart), and the movement of a lung tumor becomes more complex as it shifts from a linear to loop or hysteresis curve movement [6], [8]. Therefore, while tracking tumor movement at a specific point using fiducial markers, the movement of each fiducial marker must be observed.

Four-dimensional computed tomography (4DCT) with a body surface infrared marker is useful to determine the movement of a lung tumor or fiducial marker before planning SBRT [9]. The 4DCT enables the measurement of lung volumes and evaluates the relationship of respiratory movements between lung volume and lung tumors.

The respiratory gating method using a spirometer allows for tumor monitoring based on lung volume. This method also provides averaged and consistent information [3], [10] that can be used to evaluate the patient’s pulmonary function. In addition, this method can be used to safely determine the location of the tumor using information obtained from the fiducial marker [11].

We used 4DCT images to determine the correlation between respiratory movements in various fiducial marker positions and lung volumes for lung tumors. Moreover, we evaluated the effectiveness of the combined use of 4DCT images considering each method’s advantages and disadvantages. Dual monitoring that combines fiducial markers and lung volumes may avoid deteriorated accuracy caused by fiducial marker dropout and variations between marker and tumor motion. This novel tracking approach seeks to increase the safety and accuracy of lung SBRT. We believe that the results of this study will aid in the development of a novel lung SBRT method that combines diaphragm and tumor tracking.

## 2 Materials and methods

### 2.1 Patients and materials

We retrospectively analyzed 30 patients (median age, 76.7 [50–91] years) who had fiducial markers with a diameter of 1.5 mm (FMR-201CR; Olympus Co., ltd, Tokyo, Japan) implanted inside the tumor (total number of markers in all patients, 101: 4 fiducial markers, 18 patients; 4 fiducial markers and 1 dropout, 6 patients; 2 fiducial markers and 2 dropouts, 5 patients; and 1 fiducial marker and 3 dropouts, 1 patient) and who received SBRT as lung tumor treatment during 2018–2021. Table 1 demonstrates patient characteristics, including the location of the lung tumor, the median distance of the center of gravity coordinates (CGC) between the lung tumor and the fiducial marker, and the median volume of both lungs.

**Table 1:** Clinical characteristics of the patients.

**Lung region of the tumor**	**Number (patients)**	**Markers (Median: range) (number)**	**Median CGC distance between the lung tumor and fiducial markers (range) (mm)**	**Median volume of both lungs (min–max) (cm^3^)**	**Maximum motion amplitude of the respiratory movement (median: range) (mm)**	
					**Tumor**	**Marker**
RUL	5	4	2.4	2044	6.8	5.5
		(3–4)	(0.5–4.8)	(1692–3909)	(4.4–16.2)	(4.0–17.0)
LUL	8	2.5	2.7	3396	3.8	3.3
		(1–4)	(0.7–4.3)	(2159–5190)	(1.0–13.6)	(1.0–16.3)
RML	1	4	3.1	3953	26.3	26.1
			(1.1–3.6)	(3817–4148)		
RLL	12	4	2.2	2714	11.3	14.0
		(2–4)	(0.7–5.0)	(1524–5338)	(6.2–25.9)	(3.3–31.1)
LLL	4	4	3.2	3022	15.3	20.2
		(3–4)	(0.5–4.2)	(2273–6464)	(12.9–20.3)	(16.1–26.2)
Total	30	4	2.6	2940	8.9	13.4
		(1–4)	(0.5–5.0)	(1524–6464)	(1.0–26.3)	(1.0–31.1)

Pt: Patient, LUL: left upper lobe, LLL: left lower lobe, RUL: right upper lobe, RML: right middle lobe, RLL: right lower lobe, CGC: center of gravity coordinates.

This study was approved by the Ethics Committee of the Institutional Review Board of Yamaguchi University Hospital, Yamaguchi, Japan Hospital and was conducted in accordance with the ethical guidelines of the Declaration of Helsinki (IRB: H2021-055). This study is retrospective in nature and thus the requirement for informed consent was waived.

4DCT (SOMATOM Definition AS Open, Siemens AG, Munich, Germany) was performed by measuring changes in chest wall motion height using an infrared camera system and an external patient marker (Varian Medical Systems, Palo Alto, CA). Moreover, phase-based sorting was used for the data obtained from CT scans. Phase-based sorting involved dividing the breathing cycle of the acquired respiratory signal into 10 phase bins (0 %–90 %). Lung SBRT was performed using a radiation therapy device (Truebeam, Varian Medical Systems) and a real-time tumor-tracking radiotherapy system (SyncTrax, Shimadzu, Kyoto, Japan) with a motor-driven base for the gating window.

### 2.2 Automatic contour propagation for lung tumors, fiducial markers, and lung volume on 4DCT

Data related to the lung tumor and volumes (ipsilateral, contralateral, and bilateral lungs) obtained using 4DCT on a radiotherapy planning support software (MIM; Maestro, MIM Software Inc., OH, USA) were propagated using planning CT contour information obtained from a radiation oncologist. MIM was also used to propagate the fiducial markers [12]. Using MIM, the fiducial marker was automatically contoured to a structure of ≥2000 HU in the participant’s body for each 4DCT phase. Furthermore, as needed, the medical radiologist visually corrected the propagated lung tumor, lung volume, and fiducial marker, including the influence of metal artifacts. The ipsilateral, contralateral, and bilateral lung volumes were subsequently calculated for each 4DCT phase. Notably, the end-exhalation phase for each patient was defined as the phase with minimum ipsilateral and contralateral lung volumes, as calculated using 4DCT. In addition, we evaluated the difference in the minimum lung volume between the ipsilateral and contralateral lungs.

### 2.3 Calculation of the distance of the CGC between the lung tumor and fiducial markers

Positional lung tumor coordinates (anterior–posterior [APt], right–left [RLt], and super–inferior [SIt]) and fiducial marker positions (anterior–posterior [APm], right–left [RLm], and super–inferior [SIm]) were calculated on the CGCs of the contoured structure of the lung tumor, lung volume, and fiducial marker using MIM. Moreover, the distances of the CGC between the lung tumor and fiducial markers were calculated using MIM.

The respiratory movements between the lung tumor and fiducial markers on 4DCT images were normalized using the end-exhalation phase for each patient with the following formula [13]:

(1) $$\mathrm{Motion}\;\;amplitude\;\left(respiratory\;\;movement\;\;COM\;\;of\;\;the\;\;lung\;\;tumor\right)\;=\sqrt{{\left({\mathrm{AP}}_{t(ex)}-\;{\mathrm{AP}}_{t(other)}\right)}^{2}+{\left({\mathrm{RL}}_{t(ex)}-{\mathrm{RL}}_{t(other)}\right)}^{2}+{\left({\mathrm{SI}}_{t(ex)}-{\mathrm{SI}}_{t(other)}\right)}^{2}}$$

(2) $$\mathrm{Motion}\;\;amplitude\;\left(respiratory\;\;movement\;\;of\;\;the\;\;fiducial\;\;marker\right)\;=\sqrt{{\left({\mathrm{AP}}_{m(ex)}-{\mathrm{AP}}_{m(other)}\right)}^{2}+{\left({\mathrm{RL}}_{m(ex)}-{\mathrm{RL}}_{m(other)}\right)}^{2}+{\left({\mathrm{SI}}_{m(ex)}-{\mathrm{SI}}_{m(other)}\right)}^{2}}$$

where AP (ex), RL (ex), SI (ex), AP (other), RL (other), and SI (other) denote motion amplitudes in each direction based on the end-exhalation phase and other phases.

### 2.4 Cross-correlation coefficient of the phase waveform between the lung tumor and fiducial markers or lung volume

The cross-correlation coefficients of the phase waveforms between the lung tumor and fiducial markers or lung volume were calculated for all three gating windows (all phases, ≤ 2 mm^3^, and ≤ 3 mm^3^ of the motion amplitude of the lung tumor CGC normalized using the end-expiratory phase). Moreover, the cross-correlation coefficients of the lung volume were calculated using the three lung volume types (ipsilateral, contralateral, and bilateral). Notably, the cross-correlation coefficient was calculated using the following formula:

(3) $$\mathrm{Cross}\;correlation\;coefficient\left(X,Y\right)=\frac{\sum \left(x-\overset{¯}{x}\right)\sum \left(y-\overset{¯}{y}\;\right)}{\sqrt{\sum {\left(x-\overset{¯}{x}\right)}^{2}\sum {\left(y-\overset{¯}{y}\right)}^{2}}}$$

where x denotes the motion amplitude value normalized for the end-exhalation phase, and y denotes the phase value of 4DCT.

Subsequently, the ratio of ≥0.9 for the correlation coefficient of the phase waveforms between the lung tumor and fiducial markers or lung volume was calculated for each of the three gating windows. In addition, the number of advance/phase drifts in the motion amplitude of the fiducial markers and the ipsilateral, contralateral, and bilateral lung volumes for the motion amplitude of the lung tumor was calculated along with the motion amplitude of the lung tumor at the advance/phase drift.

### 2.5 Analysis of the relationship of the cross-correlation coefficient of the phase waveform between the lung tumor and fiducial markers or lung volume

The cross-correlation coefficients of fiducial markers’ positions for all phases were analyzed during the lung tumor phases of the three gating windows.

A relationship in the cross-correlation coefficient of fiducial marker positions (all and optimal fiducial markers) and lung volume (ipsilateral, contralateral, and bilateral) for the lung tumor was analyzed during the lung tumor phases of the three gating windows using the Mann–Whitney *U* test. In this context, the optimal fiducial marker was defined as the marker with the highest correlation among all markers.

By comparing the cross-correlation coefficient between the lung tumor and lung (ipsilateral, contralateral, and bilateral) volume, the number of low cross-correlation coefficients between the lung tumor and fiducial markers was calculated.

In addition, to determine whether the motion amplitude of the fiducial markers close to the lung tumor was similar to that of the lung tumor, the cross-correlation coefficients between the lung tumor and fiducial markers were correlated with the distance of the CGC between the lung tumor and the fiducial marker at the end-expiratory phase in each of the three gating windows.

Statistical significance was assessed through a nonparametric statistical hypothesis test (Mann–Whitney *U* test) using JMP Pro 15 (SAS Institute Inc., Cary, NC, USA). Results were considered significant at *p*-values of < 0.001. Moreover, the correlation coefficients (r) were determined using JMP Pro 15.

## 3 Results

Table 1 presents the clinical characteristics of the lung tumor and fiducial markers or lung volume for the three lung regions. For all patients, the median CGC distance between the lung tumor and fiducial markers was 2.6 (0.5–5.0) mm, and the median bilateral lung volume was 2941 (1524–6464) cm^3^. The maximum amplitudes of respiratory movements were 1.0–26.3 mm and 1.0–31.1 mm for the lung tumor and fiducial markers, respectively (Table 1).

**Fig. 1:**
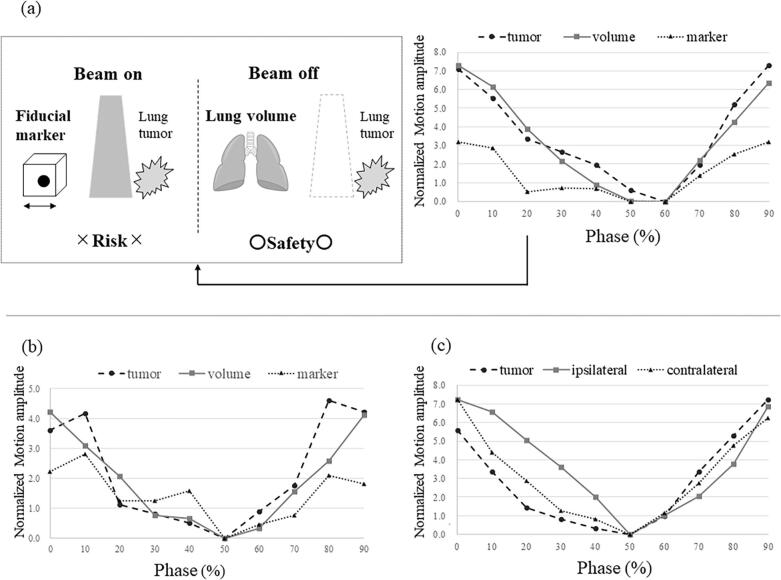
Typical phase waveforms and illustration of the lung tumor and the optimal fiducial marker or bilateral lung volume Fig. 1(a) shows the risk of beam-on by motion amplitude between the lung tumor and the fiducial marker. The graph shows a low correlation between the lung tumor and the fiducial markers and a high correlation between the lung tumor and lung volume. Fig. 1(b) shows a low correlation between the lung tumor and the fiducial marker in the upper left lung, wherein three fiducial markers were dropped out; hence, only one fiducial marker was assessed (patient information: patient with one implanted fiducial marker and three dropped fiducial markers). Fig. 1(c) shows the case of different correlations between the lung tumor and the ipsilateral or contralateral lung. The vertical axis shows the motion amplitude normalized for the end-expiratory phase. The horizontal axis shows the 4DCT phase.

Fig. 1 shows the typical phase waveforms and illustrates the lung tumor and fiducial markers. Fig. 1(a) presents the risk of unplanned irradiation (lung tumor outside the gating window and fiducial markers within the gating window) using fiducial markers and the safety of normal beam-off according to the lung volume. The graph demonstrates a low correlation between the lung tumor and fiducial markers and a high correlation between the lung tumor and lung volume. Fig. 1(b) shows a low correlation between the lung tumor and fiducial markers in the upper left lung, wherein three fiducial markers dropped out, leaving only one marker. Fig. 1(c) shows the difference in the correlation between the lung tumor and ipsilateral and contralateral lung volumes.

**Fig. 2:**
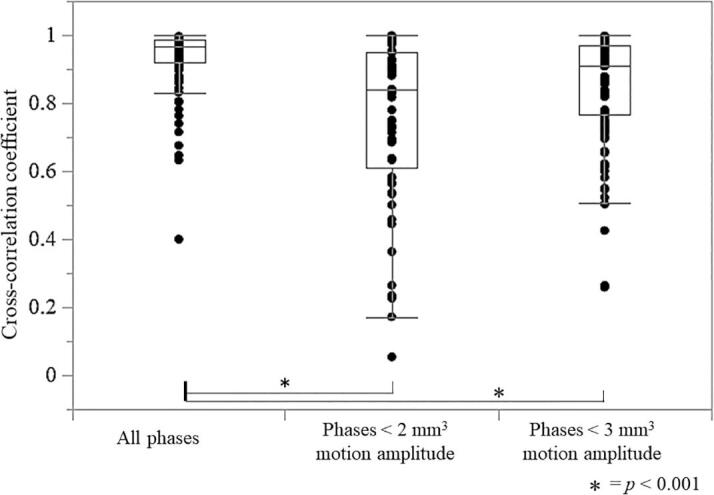
Cross-correlation coefficient of the fiducial markers for changes in three gating windows (all phases and ≤2 mm^3^ and ≤3 mm^3^ of the phase for lung tumor motion amplitude) The cross-correlation coefficient of the fiducial markers of all phases shows a significant difference between the ≤2 mm^3^ and ≤3 mm^3^ gating windows using the Mann–Whitney *U* test (*p* < 0.001).

The cross-correlation coefficient of the fiducial marker in all phases (median: 0.98) significantly differed between the ≤2 mm^3^ (median: 0.85) and ≤3 mm^3^ (median: 0.88) gating windows (Fig. 2).

**Fig. 3:**
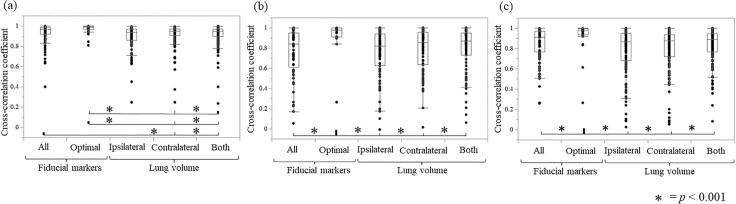
Cross-correlation coefficient of the phase waveform between the lung tumor and fiducial markers (all fiducial markers and the optimal fiducial marker) or lung volume (ipsilateral, contralateral, and bilateral) (a). All phases, (b) ≤2 mm^3^ gating phase for lung tumor motion amplitude, and (c) ≤3 mm^3^ gating phase for lung tumor motion amplitude. A significant difference is found in the correlation coefficient between the optimal fiducial marker and the ipsilateral lung, contralateral lung, and bilateral lung volumes across all three phases.

The median cross-correlation coefficients of the phase waveform between the lung tumor and all fiducial markers, optimal fiducial markers, ipsilateral lung volume, contralateral lung volume, and bilateral lung volume were 0.97, 0.99, 0.93, 0.95, and 0.94, respectively, in all phases; 0.84, 0.98, 0.82, 0.86, and 0.87, respectively, in the ≤2 mm^3^ gating window; and 0.91, 0.98, 0.87, 0.88, and 0.89, respectively, in the ≤3 mm^3^ gating window (Table 2). The proportion of high cross-correlation coefficients was lower for all markers and lung volumes than for optimal fiducial markers (Table 2). A significant difference was found in the cross-correlation coefficient between the optimal fiducial marker and the ipsilateral, contralateral, and bilateral lung volumes across all three phases (*p*-value < 0.001) (Fig. 3).

**Table 2:** Cross-correlation coefficient of the phase waveform between the lung tumor and fiducial markers or lung volume.

**Phase**	**Cross-correlation coefficient**	**Fiducial marker**		**Lung volume**		
		**All fiducial markers**	**Optimal fiducial marker**	**Ipsilateral lung**	**Contralateral lung**	**Both lungs**
All-phase	Median	0.97	0.99	0.93	0.95	0.94
	(min)	(0.40)	(0.81)	(0.25)	(0.25)	(0.24)
	Ratio > 0.9	79.2 %	93.8 %	56.7 %	70.9 %	66.0 %
Phase ≤ 2 mm^3^	Median	0.84	0.98	0.82	0.86	0.87
	(min)	(0.05)	(0.26)	(0.01)	(0.02)	(0.06)
	Ratio > 0.9	38.5 %	82.6 %	34.7 %	44.2 %	38.5 %
Phase ≤ 3 mm^3^	Median	0.91	0.98	0.87	0.88	0.89
	(min)	(0.26)	(0.26)	(0.25)	(0.05)	(0.08)
	Ratio > 0.9	52.6 %	86.1 %	39.9 %	40.6 %	44.6 %

Regarding the comparison of the waveforms between the lung tumor and lung (ipsilateral, contralateral, and bilateral) volume, the numbers of low correlations between the lung tumor and fiducial markers were 28, 28, and 30, respectively, in the ≤2 mm^3^ gating window; 38, 33, and 35, respectively, in the ≤3 mm^3^ gating window; and 38, 34, and 40, respectively, among all-phase waveforms. Approximately 30 % of the fiducial markers demonstrated low correlations between the lung tumor and fiducial markers and high correlations between the lung tumor and lung volumes.

End-exhalation phases were defined as the 40 %, 50 %, and 70 % phases in 5, 20, and 5 patients, respectively. Notably, 15 patients demonstrated different minimum ipsilateral and contralateral lung volumes. The differences in the motion amplitude by varying normalized settings in the 40 % and 50 % phases were 0.42 mm and 0.80 mm, respectively.

The distances of the CGC between the lung tumor and fiducial markers were not correlated with the cross-correlation coefficient for the waveform between the lung tumor and all fiducial markers in all three gating windows (all phases: r = −0.08, ≤2 mm^3^ gating window: r = −0.02, and ≤3 mm^3^ gating window: r = −0.05) (Supplementary material).

## 4 Discussion

This study evaluated the correlation between the fiducial marker movement near the tumor and changes in lung volume. In addition, we reviewed the advantages and disadvantages of each monitoring type and the effectiveness of dual amplitude-gated SBRT using fiducial markers and lung volume.

As shown in Table 2, all three gating windows demonstrated high cross-correlation coefficients for the optimal fiducial marker. Notably, the proportion of high cross-correlation coefficients (r > 0.9) for the ≤2 mm^3^ gating window was the same (38.5 %) for all fiducial markers and bilateral lung volumes. However, it was 82.6 % when the optimal fiducial marker was selected. Selecting the fiducial marker with the highest cross-correlation coefficient helps avoid unplanned irradiation and prolonged treatments, as required when the fiducial marker is outside the gating window or when the lung tumor is within the gating window. As shown in Fig. 1(c), the low cross-correlation coefficient of the ipsilateral lung volume may extend the treatment time because of the residual tumor motion in the gating window. Widening the gating window can help avoid treatment prolongation; however, it increases the risk of unplanned irradiation. Therefore, we consider lung volume monitoring with a high cross-correlation coefficient as a safe practice to avoid unplanned irradiation.

The CGC distance between the lung tumor and fiducial markers were found no correlation. The slight differences in positional coordinates between the lung tumor and fiducial markers were likely caused by complicated respiratory movements, including linear movement and looping and hysteresis curves [6]. Therefore, determining each patient’s optimal fiducial marker is important for accurately tracking the lung tumor and maximizing treatment quality using pretreatment 4DCT and 4D magnetic resonance imaging [9], [14].

In all three gating windows, the cross-correlation coefficients between the lung tumor and the optimal fiducial marker differed significantly from those between the lung tumor and lung volume (Fig. 3). Notably, the correlation coefficient of the optimal fiducial marker was 0.99, and the median correlation coefficient of the volume of both lungs was slightly inferior at 0.94. This may be attributable to differences in the end-expiratory phase due to lung function between ipsilateral and contralateral lungs and changes in respiratory volume due to 4DCT from the waveform represented by the body surface marker [1], [15]. The correlation between the lung tumor and fiducial markers was low, with approximately 30 % of the fiducial markers obtained by comparison between lung tumor and lung volume. Therefore, the lung volume cannot completely track slight respiratory movements; however, it is effective for stable and auxiliary monitoring [16].

Differences in the minimum lung volume between the ipsilateral and contralateral lungs were observed in 15 of 30 patients. In addition, the motion amplitude difference obtained by varying the normalized setting of 50 % phase was 0.8 mm, and the maximum difference was 3.3 mm for the included patients. These motion amplitude differences are attributable to individual differences as well as to differences in movements between the body surface infrared marker and the actual lung volume, delayed transmission of respiratory information, and other causes [15]. When performing dual amplitude-gated SBRT using lung volumes and fiducial markers, phase shifts should be considered on a case-by-case basis.

As shown in Fig. 1(b), the fiducial marker is tracked at a point near the lung tumor, making it possible to track its movement in a manner similar to complicated lung tumor movement tracking. Lung tumors can be reliably tracked by monitoring the lung volume. Fig. 1(a) shows the case of a patient in whom two of the three fiducial markers were dropped out, and the correlation between the fiducial markers was low at 0.27. There was a risk of subsequent dropout with treatment using fiducial markers alone [7]. Hence, in such cases, we believe that lung volume monitoring is an effective secondary method that could be used as a backup during treatment.

This study has three possible limitations. First, the small sample size (30) and the different number of markers for each patient led to uncertainty in the research results regarding the numerical values, such as the phase shift value of motion amplitude, because of a phase drift. Second, differences in lung tumor movement due to lung lesions were not assessed. Third, despite careful verification, the calculated 4DCT reconstruction errors due to irregular breathing patterns may not be accurate between the 4DCT scan and the actual treatment [17], [18]. Therefore, the appropriateness of 4DCT for measuring irregular breathing patterns should be verified before clinical use. Slightly irregular movement of the tumor and marker could be verified using the correlation of the average stable lung volume waveform. In addition, there may be limitations unknown at this time. Future studies should incorporate the Jacoby analysis of lung respiration using 4DCT for more-accurate risk prediction and assessment during the treatment period [19]. We believe that our findings will aid the development of new MRI-guided markerless lung SBRT methods by combining diaphragm and tumor tracking.

In this study, we evaluated the risks and advantages of fiducial marker monitoring by points and lung volume monitoring. Lung volume monitoring was identified as an effective secondary method. We found that pretreatment 4DCT evaluation helps improve the assessment of respiratory movements and the development of safety motion management for lung SBRT.

## Declaration of Competing Interest

The authors declare that they have no known competing financial interests or personal relationships that could have appeared to influence the work reported in this paper.

## References

[1] Brandner E.D., Chetty I.J., Giaddui T.G., Xiao Y., Huq M.S. (2017). Motion management strategies and technical issues associated with stereotactic body radiotherapy of thoracic and upper abdominal tumors: a review from NRG oncology. Med Phys.

[2] Bertholet J., Knopf A., Eiben B., McClelland J., Grimwood A., Harris E. (2019). Real-time intrafraction motion monitoring in external beam radiotherapy. Phys Med Biol.

[3] Keall P.J., Mageras G.S., Balter J.M., Emery R.S., Forster K.M., Jiang S.B. (2006). The management of respiratory motion in radiation oncology report of AAPM Task Group 76. Med Phys.

[4] Jaccard M., Champion A., Dubouloz A., Picardi C., Plojoux J., Soccal P. (2019). Clinical experience with lung-specific electromagnetic transponders for real-time tumor tracking in lung stereotactic body radiotherapy. Phys Imaging Radiat Oncol.

[5] Hiroshima Y., Tamaki Y., Sawada T., Ishida T., Yasue K., Shinoda K. (2022). Stereotactic body radiotherapy for stage I lung cancer with a new real-time tumor tracking system. Anticancer Res.

[6] Seppenwoolde Y., Shirato H., Kitamura K., Shimizu S., van Herk M., Lebesque J.V. (2002). Precise and real-time measurement of 3D tumor motion in lung due to breathing and heartbeat, measured during radiotherapy. Int J Radiat Oncol Biol Phys.

[7] Kothary N., Heit J.J., Louie J.D., Kuo W.T., Loo B.W., Koong A. (2009). Safety and efficacy of percutaneous fiducial marker implantation for image-guided radiation therapy. J Vasc Interv Radiol.

[8] Stowe H., Ogake S., Sharma S., Kelly S., McDonald M., Stanley K. (2019). Improved respiratory motion tracking through a novel fiducial marker placement guidance system during electromagnetic navigational bronchoscopy (ENB). Radiat Oncol.

[9] Tanabe Y., Eto H. (2022). Evaluation of patient-specific motion management for radiotherapy planning computed tomography using a statistical method. Med Dosim.

[10] Wong J.W., Sharpe M.B., Jaffray D.A., Kini V.R., Robertson J.M., Stromberg J.S. (1999). The use of active breathing control (ABC) to reduce margin for breathing motion. Int J Radiat Oncol Biol Phys.

[11] Takemoto S., Shibamoto Y., Hashizume C., Miyakawa A., Murai T., Yanagi T. (2021). Changes in pulmonary function and their correlation with dose–volume parameters in patients undergoing stereotactic body radiotherapy for lung cancer. J Radiat Res.

[12] Zhang A., Li J., Qiu H., Wang W., Guo Y. (2017). Comparison of rigid and deformable registration through the respiratory phases of four-dimensional computed tomography image data sets for radiotherapy after breast-conserving surgery. Medicine.

[13] Tanabe Y., Ishida T., Eto H., Sera T., Emoto Y. (2019). Evaluation of the correlation between prostatic displacement and rectal deformation using the dice similarity coefficient of the rectum. Med Dosim.

[14] Perkins T., Lee D., Simpson J., Greer P., Goodwin J. (2021). Experimental evaluation of four-dimensional magnetic resonance imaging for radiotherapy planning of lung cancer. Phys Imaging Radiat Oncol.

[15] Gefter W.B., Lee K.S., Schiebler M.L., Parraga G., Seo J.B., Ohno Y. (2021). Pulmonary functional imaging: part 2-state-of-the-art clinical applications and opportunities for improved patient care. Radiology.

[16] Prado A., Zucca D., De la Casa M.Á., Martí J., Alonso L., de Acilu P.G. (2022). Intrafraction target shift comparison using two breath-hold systems in lung stereotactic body radiotherapy. Phys Imaging Radiat Oncol.

[17] Keikhai Farzaneh M.J.K., Momennezhad M., Naseri S. (2021). Gated radiotherapy development and its expansion. J Biomed Phys Eng.

[18] Delombaerde L., Petillion S., Weltens C., Depuydt T. (2021). Intra-fraction motion monitoring during fast modulated radiotherapy delivery in a closed-bore gantry linac. Phys Imaging Radiat Oncol.

[19] Antony R., Lonski P., Ungureanu E., Hardcastle N., Yeo A., Siva S. (2020). Independent review of 4DCT scans used for SABR treatment planning. J Appl Clin Med Phys.

