# Supplementary material for: Statistical evaluation of the effectiveness of dual amplitude-gated stereotactic body radiotherapy using fiducial markers and lung volume
Source: Phys Imaging Radiat Oncol. 2022 Oct 6;24:82–7. doi: 10.1016/j.phro.2022.10.001 (PMC9576976; doi:10.1016/j.phro.2022.10.001)
Supplement: Supplementary data 1 [file mmc1.pdf]

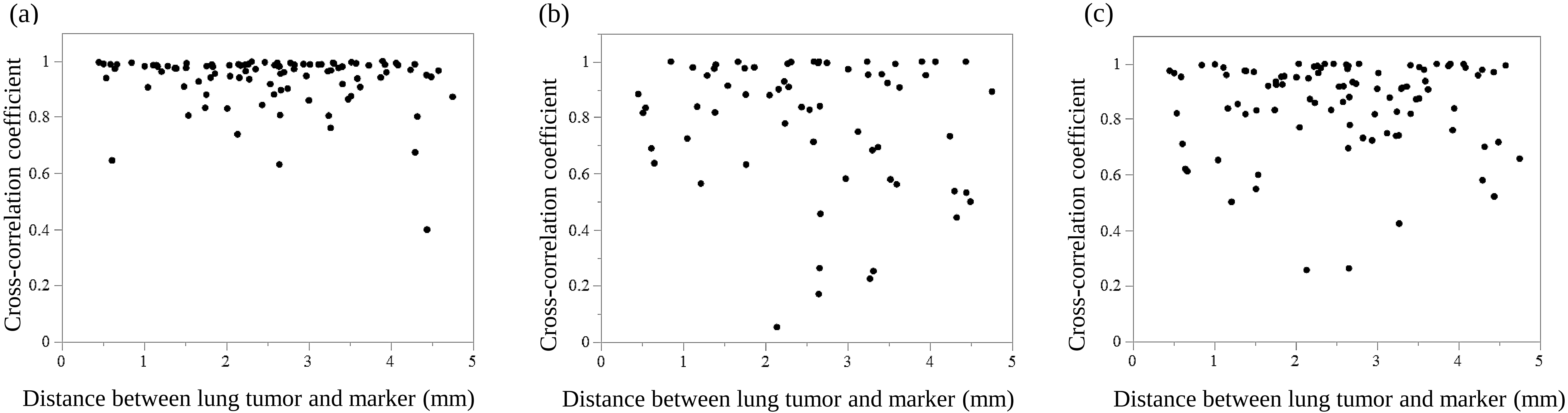

Figure S1. Relationship of the distance between the lung tumor center of gravity coordinates with the fiducial marker. (a). All phases, (b) ≤2 mm^3 gating phase with lung tumor motion amplitude, and (c) ≤3 mm^3 gating phase with lung tumor motion amplitude. No correlation is found for the distance between the lung tumor and fiducial markers across all three phases.
